# Urothelial Bladder Carcinoma in Young and Elderly Patients: Pathological Insights and Age-Related Variations

**DOI:** 10.3390/cancers17050845

**Published:** 2025-02-28

**Authors:** Andrada-Claudia Tătar, Andrada Loghin, Adela Nechifor-Boilă, Andrada Raicea, Maria-Cătălina Popelea, Călin Chibelean, Raul-Dumitru Gherasim, Angela Borda

**Affiliations:** 1 Histology Department, George Emil Palade University of Medicine, Pharmacy, Science, and Technology of Targu Mures, 540142 Targu Mures, Romania; andrada-claudia.tatar@umfst.ro (A.-C.T.); adela.nechifor-boila@umfst.ro (A.N.-B.); andrada.raicea@umfst.ro (A.R.); popelea.maria-catalina.24@stud.umfst.ro (M.-C.P.); angela.borda@umfst.ro (A.B.); 2 Doctoral School of Medicine and Pharmacy, George Emil Palade University of Medicine, Pharmacy, Science, and Technology of Targu Mures, 540142 Targu Mures, Romania; 3 Pathology Department, Mures Clinical County Hospital, 540011 Targu Mures, Romania; 4 Urology Department, George Emil Palade University of Medicine, Pharmacy, Science, and Technology of Targu Mures, 540142 Targu Mures, Romania; calin.chibelean@umfst.ro (C.C.); raul-dumitru.gherasim@umfst.ro (R.-D.G.); 5 Urology Department, Mures Clinical County Hospital, 540011 Targu Mures, Romania; 6 Pathology Department, Targu Mures Emergency Clinical County Hospital, 540136 Targu Mures, Romania

**Keywords:** bladder cancer, urothelial carcinoma, young patients, pathological characteristics

## Abstract

Background/Objectives: UBC demonstrates a relatively low prevalence in patients under 50 years old. Our study aimed to compare the pathological characteristics of UBC in young and elderly patients and to assess the age-related variations in these characteristics. Methods: This retrospective study from January 2018 to December 2022 encompassed 762 patients with an initial diagnosis of UBC. To evaluate the pathological characteristics of UBC in young patients, compared to elderly patients, we divided our cohort using a cut-off age of 50 years. Additionally, to evaluate the age-dependent variations in these characteristics, we further stratified our patients into three age groups (≤50, 51–70, and >70 years old). Results: Of the 762 patients included in our study, 37 (4.86%) were young (≤50 years old), with a median age of 44 and a M:F ratio of 3.11:1. Conventional UC was the most common histology (86.5%). Regarding tumour grade, we noticed a higher prevalence of PUNLMP (10.8% versus 2.3%) and LGPUC (45.9% versus 37.1%) and a lower prevalence of HGPUC (43.2% versus 60%) and CIS (0% versus 0.6%) in young patients (*p* = 0.008). In this group, the pTa stage revealed a higher prevalence (64.9% versus 46.8%), while pT1 and pT2 showed a lower prevalence (32.1% versus 18.9% and 20.6% versus 16.2%) (*p* = 0.179). All results remained consistent when the cohort was stratified into three age groups, showing age-related variations (*p* = 0.001 and *p* = 0.089, respectively). Conclusions: In young patients, UBC tends to be of a lower grade and stage compared to elderly patients, and these characteristics demonstrate a gradual age-related stratification.

## Simple Summary

Urothelial bladder carcinoma (UBC) exhibits a relatively low prevalence in individuals under 50. We aimed to compare the pathological features of UBC in young and elderly patients and to determine their age-dependent variations. Our study included 762 patients diagnosed with UBC between January 2018 and December 2022. We divided our cohort using a cut-off age of 50 years old and further stratified the patients into three age groups: ≤50, 51–70, and >70 years. A total of 37 (4.86%) patients were young (≤50 years old), with 86.5% of them presenting conventional UC. We noticed a higher prevalence of PUNLMP and LGPUC and a lower prevalence of HGPUC and CIS in young patients. In this group, the pTa stage was more frequent, while pT1 and pT2 were less frequent. Our results highlight the gradual age-dependent variations in these pathologic characteristics, with UBC being of a lower grade and stage in young patients.

## 1. Introduction

Bladder cancer represents the 9th most common malignancy worldwide, with more than 600,000 new cases diagnosed every year [1]. Urothelial bladder carcinoma (UBC) accounts for most of these malignancies, but less common types include squamous cell carcinoma, adenocarcinoma, and small-cell carcinoma [2]. UBC shows a peak incidence during the 8th decade of life and has a four times higher incidence rate in male patients, compared to their female counterparts [3,4]. The majority of diagnosed UBCs are conventional UCs. However, squamous, glandular, or trophoblastic differentiations are reported in varying proportions. Other histological subtypes include nested, micropapillary, plasmacytoid, and sarcomatoid, as well as combinations of these [5,6]. Most UBCs are diagnosed at a superficial, non-muscle-invasive stage (pTa, pT1, pTis), but around 15–30% of the cases are diagnosed at a muscle-invasive (pT2) or more advanced stage (pT3, pT4) [2].

A low incidence of UBC, varying between 0.8% and 3.3%, is found in patients aged under 50 years old [7]. There are controversies in defining the “young” population, as different researchers have used age cut-offs of 20, 30, 40, or 50 years old [8,9,10]. However, oncological experts define patients under 50 years old as “young” and patients under 40 years old as “very young” [11]. Many studies identified more favourable morphologic characteristics in the young cohort, primarily attributed to lower tumour grading and staging, thus validating their prognostic significance [3,12]. However, several studies show similar clinical behaviour and prognosis in the young- and old-age cohorts [8,13,14]. Owing to these conflicting opinions, as well as to the limited data in the literature, the complete picture of the disease in the young-age population is not yet completely understood.

The aim of our study was to assess the pathological characteristics of UBC in young patients (≤50 years old) and to compare them to those of elderly patients (>50 years old). As a second objective, we evaluated the age-dependent variation the in pathological characteristics of UBC by stratifying patients in three age categories (≤50, 51–70, and >70 years old).

## 2. Material and Methods

### 2.1. Study Design

**Figure 1:**
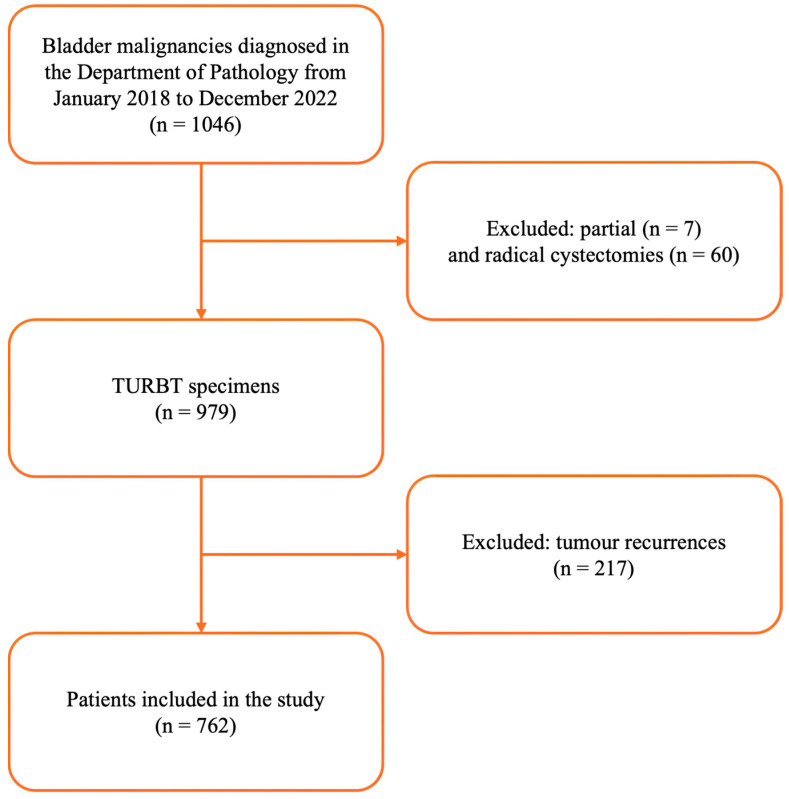
PRISMA-like flow chart for patients’ inclusion and exclusion criteria. TURBT: transurethral resection of the bladder tumour.

In this large retrospective cross-sectional single-centre study, we included patients with UBC from the Department of Urology, Clinical County Hospital, Târgu Mureș, Romania, between January 2018 and December 2022. Our study focused on patients’ initial pathological diagnosis on the first transurethral resection of the bladder tumour (TURBT). Data were retrieved from the institutional database and original pathological reports from the Pathology Department of Mureș Clinical County Hospital, Târgu Mureș, Romania. Patients with tumour recurrence and partial or radical cystectomy following the initial diagnosis were excluded from this study. The focus on the initial diagnosis ensures a more reliable analysis, as subsequent treatment options, including cystectomy, and the follow-up of recurrences are significantly influenced by patient compliance and individual treatment decisions. Figure 1 illustrates the algorithm for the inclusion and exclusion of the patients into our analysis.

Our study received ethical approval from the Ethics Committee of George Emil Palade University of Medicine, Pharmacy, Science, and Technology of Târgu Mureș (letter of approval no. 1998/5 January 2023).

### 2.2. Demographic and Pathological Data

The following demographic and pathological characteristics were evaluated: (1) age at diagnosis, (2) gender, (3) tumour histological type (conventional UC, divergent differentiation, or histological subtypes), (4) growth pattern (papillary or/and infiltrative), (5) tumour pathological grade (papillary urothelial neoplasms of low malignant potential (PUNLMP), low-grade papillary urothelial carcinoma (LGPUC) or high-grade papillary urothelial carcinomas (HGPUC)), and the (6) tumour pathological stage, including carcinoma in situ. Tumours with an infiltrative component were considered high-grade urothelial carcinomas of various stages (pT1-pT2) [15]. All cases were examined by an experienced uropathologist (AL). Histological type and tumoral grade were assessed in line with the 4th [16] and 5th [5] edition of the WHO Classification of Urinary and Male Genital Tumours. The pathological stage was established according to the 8th edition of the American Joint Committee on Cancer (AJCC) Cancer Staging Manual [17].

### 2.3. Statistical Analysis

Descriptive statistical analysis was performed using the Statistical Package for Social Sciences (SPSS, version 26, Chicago, IL, USA). Data were expressed as nominal or quantitative variables. The nominal variables were characterized by means of frequencies—n (%) and were compared using Fisher’s exact test. Continuous variables were assessed using histograms and the Kolmogorov–Smirnov test and were presented as medians (Q25-Q75). The statistical significance level was set at α = 0.05.

To assess the differences in pathological characteristics between young and elderly patients with UBC, we divided our cohort into two study groups: young (≤50 years old) and elderly (>50 years old) patients. While older studies have typically set age cut-offs at 30 or 40 years, we chose to expand our cut-off to 50 years. This decision is influenced by the fact that the pathological staging of other tumours, such as thyroid carcinoma [17], has increased its cut-off age due to better prognostic outcomes and different biological behaviours in younger patients. Additionally, advancements in medicine and rising life expectancy further support the rationale for adopting a 50-year cut-off in our study. Furthermore, to evaluate the age-dependent variation in the pathological characteristics of UBC, we stratified our cohort into three study groups: group 1 (≤50 years old), group 2 (51–70 years old), and group 3 (>70 years old), and data were analyzed in comparison.

## 3. Results

### 3.1. Patients’ Characteristics

From a total of 1046 patients, a cohort of 762 patients was included in our study, with a median age of 69 (62; 76) years. There were 37 (4.86%) patients younger than 50 years old, 381 (50%) were between 51 and 70 years old, and 344 (45.14%) were older than 70 years old. There were 606 (79.52%) males and 156 (20.48%) females, with a male/female (M:F) ratio of 3.88:1.

### 3.2. Pathological Characteristics of the Study Cases

The pathological characteristics of the study cases are summarized in Table 1. Most of the tumours were conventional UCs (n = 653, 85.7%), followed by cases with divergent differentiation (squamous, glandular, and trophoblastic) (n = 76, 9.97%) and various histological subtypes of UC (nested, micropapillary, plasmacytoid, and sarcomatoid) (n = 23, 3.02%). Eight (1.05%) cases revealed mixed histology (squamous and glandular, squamous and sarcomatoid, and glandular and micropapillary, respectively).

Of the 762 cases, 21 (2.76%) were PUNLMP, 286 (37.53%) were LGPUC, 451 (59.19%) were HGPUC. Four (0.52%) cases were not associated with a papillary component and revealed only carcinoma in situ (CIS). Regarding the 451 (59.19%) HGPUCs, 56 (12.42%) were non-invasive, and 395 (87.58%) were invasive.

Non-invasive (n = 367, 48.16%) and invasive (n = 395, 51.84%) urothelial tumours were present in approximately equal proportions. Most tumours were pTa stage (n = 363, 47.64%), 240 (31.5%) cases were pT1, 155 (20.34%) cases were pT2, and 4 (0.52%) cases were pTis.

### 3.3. Gender Distribution Among Different Tumour Types

PUNLMPs and LGPUCs were more prevalent in women (5.1% vs. 2.1% and 44.9% vs. 35.6%, respectively), while HGPUCs were more frequent in men (61.7% vs. 49.4%) (*p* = 0.018). CIS revealed similar prevalences in female and male patients (0.6% and 0.5%).

With regard to the tumour stage, pTa was more common in women (56.4% vs. 45.4%), while pT2 was more common in men (22.6% vs. 11.5%) (*p* = 0.014). The prevalence of pT1 was similar between genders (31.4% in women and 31.5% in men).

### 3.4. Demographic and Pathological Characteristics in Young Versus Elderly Patients

Of the 762 patients, 37 (4.86%) were 50 years old or younger, with a median age of 44 (42–49), and 725 (95.14%) were older than 50 years old, with a median age of 70 (63–77), respectively. The M:F ratio was 3.11:1 in young patients and 3.93:1 in elderly patients (*p* = 0.552).

Divergent differentiation and histological subtypes of UC were observed in 5 (13.5%) young patients and in 102 (14.1%) elderly patients. Squamous differentiation was the second most common histological subtype in both groups (8.1% versus 8%). There was no significant statistical difference between the two study groups (*p* = 0.734).

**Figure 2:**
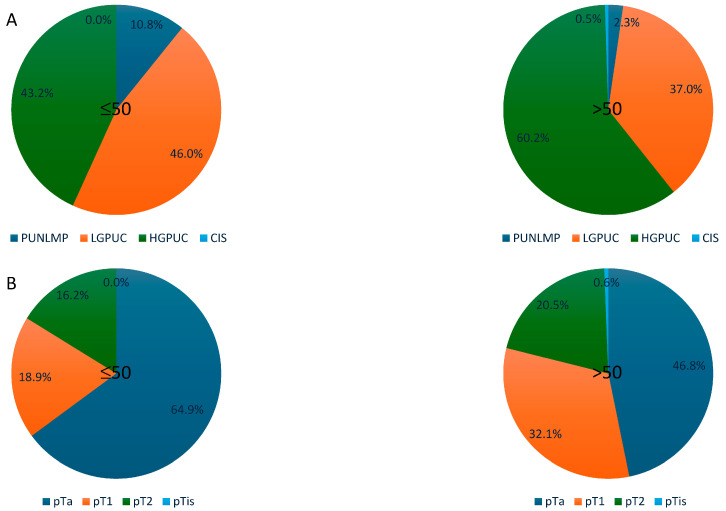
Pathological characteristics in young versus elderly patients. (**A**) Tumour grade. (**B**) Tumour stage.

We noticed a higher prevalence of PUNLMP (10.8% versus 2.3%) and LGPUC (45.9% versus 37.1%) and a lower prevalence of HGPUC (43.2% versus 60%) and CIS (0% versus 0.6%) in young patients, compared to elder patients. The differences were statistically significant (*p* = 0.008) (Figure 2A).

With regard to the tumour stage, pTa was more prevalent among young patients (64.9%) compared to elderly patients (46.8%). By contrast, the frequency of in situ (pTis) and invasive (pT1, pT2) stages was higher among elderly patients compared to young patients: pTis (0.6% versus 0%), pT1 (32.1% versus 18.9%), and pT2 (20.6% versus 16.2%). However, the differences were not statistically significant (*p* = 0.179) (Figure 2B).

### 3.5. Demographic and Pathological Characteristics in the Three Age Groups

Of the 762 patients, 37 (4.86%) belonged to group 1 (50 years old and younger)—median age 44 (42; 49), 381 (50%) to group 2 (51–70 years old)—median age 64 (59; 67.5), and 344 (45.14%) to group 3 (>70 years old)—median age 77 (74; 81). The M:F ratio was 3.11:1 in group 1, 3.4:1 in group 2, and 4.73:1 in group 3, respectively. Although the differences were not statistically significant (*p* = 0.167) between the three study groups, our data reflected that the M:F ratio increases with age, resulting in a decreased prevalence of UBC among elderly women.

Divergent differentiation and histological subtypes of UC were observed in 5 (13.5%) patients from group 1, 47 (12.3%) patients from group 2, and in 55 (16%) patients from group 3, also revealing no significant statistical differences between the three study groups (*p* = 0.080).

**Figure 3:**
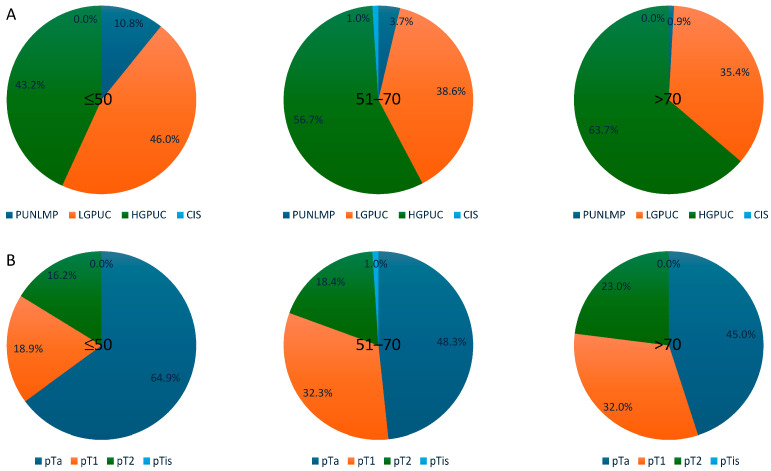
Pathological characteristics in the three age groups. (**A**) Tumour grade. (**B**) Tumour stage.

Regarding tumour grading, our data demonstrated that low-grade tumours (PUNLMPs and LGPUCs) had the highest prevalence (10.8% and 45.9%, respectively) in group 1 (young patients); their prevalence decreased in group 2 (3.7% and 38.6%, respectively) and was the lowest in group 3 (0.9% and 35.5%, respectively). By contrast, HGPUC had the highest prevalence in group 3 (63.7%) compared to both group 2 (56.7%) and group 1 (43.2%), respectively. Furthermore, the differences were statistically significant (*p* = 0.001). The four patients with CIS belonged to group 2 (0.5%) (Figure 3A).

Regarding the tumour stage, pTa revealed the highest prevalence in patients from group 1 (64.9%) and decreased in frequency for each of the other two groups (48.3% and 45,1%, respectively). By contrast, pT1 and pT2 tumour stages were the least common among patients from group 1 (18.9% and 16.2%), and their prevalence increased among patients from group 2 (32.3% and 18.4%) and group 3 (32% and 23%), respectively (*p* = 0.089) (Figure 3B).

Our results reveal that younger patients tend to present with lower grade and stage tumours, suggesting a potentially less aggressive disease course at initial diagnosis. However, further research is needed to understand the underlying biological, genetic, and environmental factors that result in increasing tumour aggressiveness over time in elderly patients. Unravelling these mechanisms could provide valuable insights into disease evolution and inform personalized management strategies.

## 4. Discussion

UBC is a rare entity in young patients and exhibits distinct pathological characteristics compared to their elderly counterparts. Relatively few studies have focused exclusively on describing the unique features of UBC in young individuals [14,18,19,20,21,22], while some have directly compared UBC in young versus elderly patients [23,24,25,26,27,28], revealing potential pathological differences between the two groups. Given the limited literature data available, the complete pathological profile of UBC in young patients remains incompletely understood.

In the present study, we aimed to compare the pathological characteristics of UBC in young versus elderly patients. To achieve this objective, we divided our study patients into two study groups, with the cut-off age of 50 years old. Furthermore, we aimed to evaluate the age-dependent variation in the pathological characteristics of UBC. For this second objective, we assigned the patients into three study groups: ≤50, 51–70, and >70 years old, respectively.

In our study, 37 patients were young (50 years old or below), accounting for only 4.86% of our cohort. A similar low prevalence of UBC (7.03%) was noticed by F. Janisch et al. in patients aged 50 or below [11].

Our study confirmed the male predominance of UBC, similar to that reported by S. Mehmood et al. in a study published in 2022 (M:F ratio of 4.5:1) [3]. Moreover, we identified a lower M:F ratio in young compared to elderly patients The gradual increase in this ratio was highlighted when we assigned the patients into three groups. A similar increase in the M:F ratio was reported by W. Zhi-hua et al. when dividing the patients into ≤40 and >40 years old groups (3.32:1 versus 6.45:1), stating the accumulation and preferential action of carcinogens in male patients [26].

We found that conventional UC was the most common in both young and elderly patients, with comparable proportions between the two groups. Similar results were published by H. S. Talwar et al., showing that conventional UC was the most common histology in both ≤40 (87.9%) and >40 (84.3%) years old patients, with similar prevalences between the two groups [2]. We observed that the prevalence of the histological subtypes of urothelial carcinoma was similar in both young and elderly patients, recognizing that age alone may not be a determining factor in the type of histological subtype present. However, when compared to conventional UC, these specific histological subtypes exhibited a worse clinical profile, being more frequently associated with distant metastases [6], indicating increased tumour aggressiveness. Our study demonstrated that squamous differentiation was the second most common histological subtype. In line with our results, S. Talwar et al. also reported squamous differentiation as the most frequent divergent differentiation, with similar prevalences between their study two groups (≤40 years old—10.3% and >40 years old—12.4%, respectively) [2].

Important differences regarding pathological grade and stage were observed in young compared to elderly patients. When patients were divided into two groups using an age cut-off of 50 years old, we noticed a higher prevalence of low-grade tumours (PUNLMP and LGPUC) and a lower prevalence of HGPUC and CIS in young patients compared to elderly patients (*p* = 0.008). O. Telli et al. also demonstrated that PUNLMP has a higher prevalence in patients <40, compared to those >40 years old (28.6% versus 16.9%) [27]. Several studies have consistently reported a higher prevalence of LGPUC (varying from 48.7% to 80.5%) and a lower prevalence of HGPUC (varying from 19.3% to 51.3%) in patients ≤40 years old, compared to older patients [23,24,25]. Moreover, a study conducted by C. M. de la Calle et al. on a large cohort of patients (n = 3314) demonstrated that patients ≤40 have a higher prevalence of low-grade tumours (72.7% versus 48.3%) and a lower prevalence of high-grade tumours (27.3% versus 51.7%) compared to those >40 years old [28]. Within our cohort of young patients, the most frequent histological grade was LGPUC. Other authors have also reported that LGPUC has a high prevalence in patients <40 years old (ranging from 50% to 93.5%), confirming that this histological grade is the most common among this population [3,19,20,21].

When patients were assigned into three age groups, we noticed that the high prevalence of PUNLMP and LGPUC among young patients was maintained. Additionally, there was a gradual decrease in prevalence for the other two groups (*p* = 0.001). A study published by Poletajew et al. demonstrated the same gradual decrease for PUNLMP (G1) with each study group (<41—77.8%, 41–50—30.5%, and >50—26.0%) [7]. Contrarily, HGPUC showed a low prevalence in young patients and demonstrated a gradual increase in the other two groups (*p* = 0.001). Our results are in line with the study conducted by F. Janisch et al., which also demonstrated a gradual increase in the prevalence of HGPUC with each study group (≤40—50%, 41–50—74.3% and ≥51—80.1%) [11].

Regarding the tumour stage, our study found that pTa was the most prevalent among young patients. Our findings align with previous studies, which also reported that the pTa stage is the most prevalent among patients younger than 40 years old, with prevalence rates ranging from 64.4% to 82.5% [18,19,20]. Moreover, a study published by M. Albakri et al. revealed that in patients younger than 45 years old, 84.7% of tumours were non-muscle invasive (pTa-pT1) [29]. Our results show that invasive stages (pT1 and pT2) were less prevalent in young compared to elderly patients. These findings are consistent with those of previously published studies, with a low prevalence of pT1 (from 9.5% to 19.6%) and pT2 (from 1.2% to 18.6%) in young patients [19,20,24,27,28,30].

When we assigned patients into three age groups, we found that the pTa stage presented the highest prevalence in patients ≤50 years old and the lowest prevalence in patients >70 years old. The opposite was noticed for invasive stages (pT1 and pT2), which presented the lowest prevalence in patients ≤50 years old and the highest prevalence in patients >70 years old. Similar data were reported by Poletajew et al., which demonstrated a gradual decrease in the pTa stage (<41—100%, 41–50—76.3%, >50—62.7%) and a gradual increase in pT1 (<41—0%, 41–50—11.9%, >50—19.3%) and =/> pT2 (<41—0%, 41–50—11%, >50—15.6%) with each age group [7].

While our study is centred on tumour morphology and does not include clinical correlations, the potential role of predictive markers in assessing tumour aggressiveness is an important direction for future research. Non-invasive biomarkers, such as the systemic inflammatory index [31], have shown promise in evaluating disease progression and could significantly enhance personalized therapeutic strategies and patient management. Future research should explore these aspects to complement morphological assessments and optimize patient outcomes.

The limitations of our study are represented by its retrospective nature of our observations and by the fact that our cohort was obtained from a single institution. However, the extensive number of patients included in this study over a substantial 5-year period may enhance the transferability of the findings to other populations. Furthermore, our institution is part of a prominent university centre that serves patients from different parts of the country, ensuring a broad diversity of cases.

## 5. Conclusions

In our study, distinct morphological characteristics of UBC were observed in young compared to elderly patients. In patients younger than 50 years old, UBCs had a lower M:F ratio and were mainly conventional UCs of low tumour grade and stage, whereas in elderly patients, UBCs were more frequently of high grade and corresponded generally to a more advanced tumour stage. Moreover, we found an age-dependent gradual stratification of these morphological characteristics, as the pathological grade and stage became more severe as age increased. Further studies are needed to identify and elucidate the adverse prognostic factors that lead to more severe disease in elderly patients.

## Figures and Tables

**Table 1:** Demographic and pathological characteristics of the patients included in this study.

				Total n = 762	Young Patients (≤50) n = 37 (4.86%)	Elderly Patients (>50) n = 725 (95.14%)		*p* *	*p* **
						51–70 n = 381 (50%)	>70 n = 344 (45.14%)		
Gender n (%)								0.552	0.167
Male				606 (79.52)	28 (75.68)	294 (77.17)	284 (82.56)		
Female				156 (20.48)	9 (24.32)	87 (22.83)	60 (17.44)		
Tumour grade n (%)								0.008	0.001
PUNLMP				21 (2.76)	4 (10.81)	14 (3.67)	3 (0.87)		
LGPUC				286 (37.53)	17 (45.95)	147 (38.58)	122 (35.47)		
HGPUC				451 (59.19)	16 (43.24)	216 (56.69)	219 (63.66)		
Non-invasive				56 (7.35)	3 (8.11)	23 (6.04)	30 (8.72)		
Invasive				395 (51.84)	13 (35.14)	193 (50.66)	189 (54.94)		
CIS				4 (0.52)	0 (0.00)	4 (1.05)	0 (0.00)		
Tumour stage n (%)								0.179	0.089
pTis				4 (0.52)	0 (0.00)	4 (1.05)	0 (0.00)		
pTa				363 (47.63)	24 (64.86)	184 (48.29)	155 (45.06)		
pT1				240 (31.49)	7 (18.92)	123 (32.28)	110 (31.98)		
pT2				155 (20.34)	6 (16.22)	70 (18.37)	79 (22.97)		
Histological subtype n (%)								0.734	0.080
Conventional UC				655 (85.96)	32 (86.49)	334 (87.66)	289 (84.01)		
UC with squamous differentiation				61 (8.01)	3 (8.11)	24 (6.30)	34 (9.88)		
UC with glandular differentiation				14 (1.84)	0 (0.00)	10 (2.62)	4 (1.16)		
UC with trophoblastic differentiation				1 (0.13)	0 (0.00)	1 (0.26)	0 (0.00)		
Nested UC				1 (0.13)	0 (0.00)	1 (0.26)	0 (0.00)		
Micropapillary UC				14 (1.84)	1 (2.70)	3 (0.79)	10 (2.90)		
Plasmacitoid UC				4 (0.52)	0 (0.00)	4 (1.05)	0 (0.00)		
Sarcomatoid UC				4 (0.52)	0 (0.00)	2 (0.52)	2 (0.58)		
Mixed									
Squamous + glandular				3 (0.39)	0 (0.00)	0 (0.00)	3 (0.87)		
Squamous + sarcomatoid				2 (0.26)	0 (0.00)	0 (0.00)	2 (0.58)		
Glandular + micropapillary				3 (0.39)	1 (2.70)	2 (0.52)	0 (0.00)		

* *p*-value was obtained by comparing the different prevalences between young and elderly patients; ** *p*-value was obtained by comparing the different prevalences between the three age groups. PUNLMP: papillary urothelial neoplasms of low malignant potential; LGPUC: low-grade papillary urothelial carcinoma; HGPUC: high-grade papillary urothelial carcinoma; CIS: carcinoma in situ; UC: urothelial carcinoma.

## Data Availability

Dataset available on request from the authors.
